# Limits of Defect Tolerance in Perovskite Nanocrystals: Effect of Local Electrostatic Potential on Trap States

**DOI:** 10.1021/jacs.2c02027

**Published:** 2022-06-14

**Authors:** Indy du Fossé, Jence T. Mulder, Guilherme Almeida, Anne G. M. Spruit, Ivan Infante, Ferdinand C. Grozema, Arjan J. Houtepen

**Affiliations:** † Optoelectronic Materials Section, Faculty of Applied Sciences, Delft University of Technology, Van der Maasweg 9, 2629 HZ Delft, The Netherlands; § Department of Nanochemistry, Istituto Italiano di Tecnologia, Via Morego 30, 16163 Genova, Italy

## Abstract

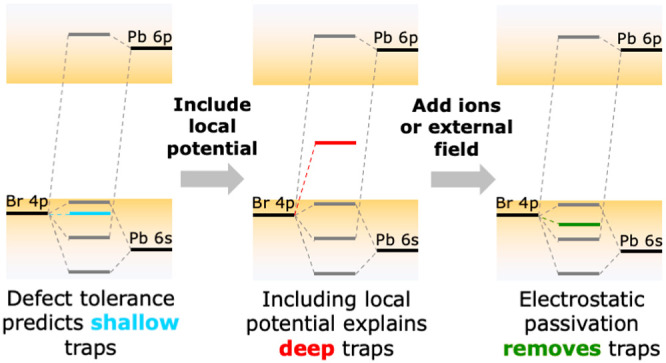

One of the most promising properties of lead halide perovskite nanocrystals (NCs) is their defect tolerance. It is often argued that, due to the electronic structure of the conduction and valence bands, undercoordinated ions can only form localized levels inside or close to the band edges (i.e., shallow traps). However, multiple studies have shown that dangling bonds on surface Br^–^ can still create deep trap states. Here, we argue that the traditional picture of defect tolerance is incomplete and that deep Br^–^ traps can be explained by considering the local environment of the trap states. Using density functional theory calculations, we show that surface Br^–^ sites experience a destabilizing local electrostatic potential that pushes their dangling orbitals into the bandgap. These deep trap states can be electrostatically passivated through the addition of ions that stabilize the dangling orbitals via ionic interactions without covalently binding to the NC surface. These results shed light on the formation of deep traps in perovskite NCs and provide strategies to remove them from the bandgap.

As a result of their high photoluminescence quantum yield, facile synthesis, narrow emission width, and tunable bandgap across the visible spectrum depending on the halide composition,^1,2^ lead halide perovskite nanocrystals (NCs) are of great interest for application in devices.^3−5^ For instance, they can be used as a color-converting phosphor,^1,6^ lasing material,^7−9^ absorber layer in solar cells,^10−13^ and emitter in light-emitting diodes.^1,6,14^ The high performance of lead halide perovskite-based materials is often linked to their defect tolerance, which is attributed to a combination of the high formation energy of defects^15,16^ and the electronic structure of the conduction (CB) and valence bands (VB).^1,2,17^ The latter point is illustrated in Figure 1, where the electronic structure of perovskites is compared with that of common “defect-intolerant” semiconductors, which include II–VI (e.g., CdSe) and III–V (e.g., InP) materials. Taking CdSe as an example, as shown in Figure 1, the bandgap is formed between bonding states (the VB) and antibonding states (the CB). As a result, nonbonding orbitals from undercoordinated atoms are likely to lie in the bandgap. This has indeed been shown to be the case for two-coordinated chalcogenides.^18−20^ However, the spherical symmetry of the s orbital of the metal ensures it is split out of the bandgap, even if the metal is undercoordinated.^18^ In lead halide perovskites (see the example of CsPbBr_3_ in Figure 1), the top of the VB consists of the antibonding interaction between the Br 4p and the Pb 6s orbitals, while the antibonding interaction between Br 4p and Pb 6p orbitals forms the CB edge. As both band edges consist of antibonding orbitals, nonbonding orbitals are expected to either lie within the bands or form shallow traps.^1,2,17^

**Figure 1:**
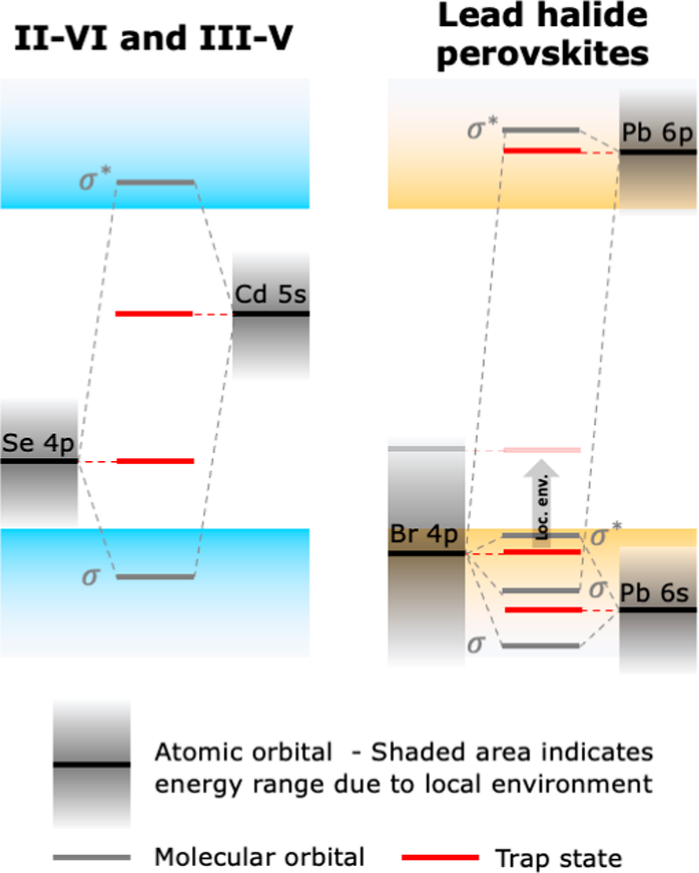
Electronic structure of (left) defect-intolerant materials, such as CdSe, and (right) defect-tolerant materials, like CsPbBr_3_. In defect-intolerant materials, the VB and CB are respectively composed of bonding and antibonding orbitals, causing nonbonding atomic orbitals (AOs, black) to form deep trap states (red). In defect-tolerant materials, both the VB and CB are formed by antibonding orbitals, so that nonbonding AOs are expected to lie close to or in the bands. However, differences in the local environment of each atom can lead to shifts of the energy of the AOs (gray-shaded areas), thus pushing trap states into the bandgap even in defect-tolerant materials, as illustrated by the gray arrow.

However, computational studies on perovskite NCs show that excess surface halide ions^16,21^ or stripping of the perovskite surface^22^ can still create deep trap states in the form of undercoordinated surface Br^–^, suggesting that the above description of defect tolerance is incomplete. In the current work, we use density functional theory (DFT) calculations on CsPbBr_3_ NCs to show that this apparent discrepancy can be understood by taking the local environment of the undercoordinated halide ions into account. Although the energy of the Br 4p orbitals lies within the VB in the perovskite bulk, a Br^–^ ion at the surface experiences a different local electrostatic potential. If the electrostatic potential is destabilizing, it can push the nonbonding Br^–^ orbitals into the bandgap; if it is stabilizing, for instance due to the presence of ionic species, dangling orbitals can be pushed further into the VB. The resulting spread in the energy of atomic orbitals (AOs) is schematically illustrated in Figure 1 by the gray-shaded areas.

From TEM images and X-ray diffraction, it is known that as-synthesized CsPbBr_3_ NCs present a cubic shape and an orthorhombic crystal structure.^23,24^ They are typically capped by oleylammonium and oleate ligands^25,26^ and have excess Br^–^ and Cs^+^ (some of which may be replaced by oleylammonium cations) at the surface.^21,22,27^ These characteristics suggest a CsBr-terminated NC, and, in line with previous computational works,^21,22,28,29^ we decided to construct a cubic Cs_324_Pb_216_Br_756_ NC model system (see Figure S1). After this step, we followed the approach of Bodnarchuk et al. (see the Supporting Information for computational details)^22^ to simulate the variation of the NC surface by the stepwise removal of the outer CsBr layer (models M1–M5, see Figure S2), followed by the gradual removal of the underlying PbBr_2_ layer (models M6–M10, see Figure 2). The highest occupied molecular orbital (HOMO) of each model is shown in Figure 2B. We further calculate the density of states (DOS), inverse participation ratio (IPR), and crystal orbital overlap population (COOP) for the Pb–Br interaction for all models, as shown in Figure 2C (see the Supporting Information for more details on these analyses).

**Figure 2:**
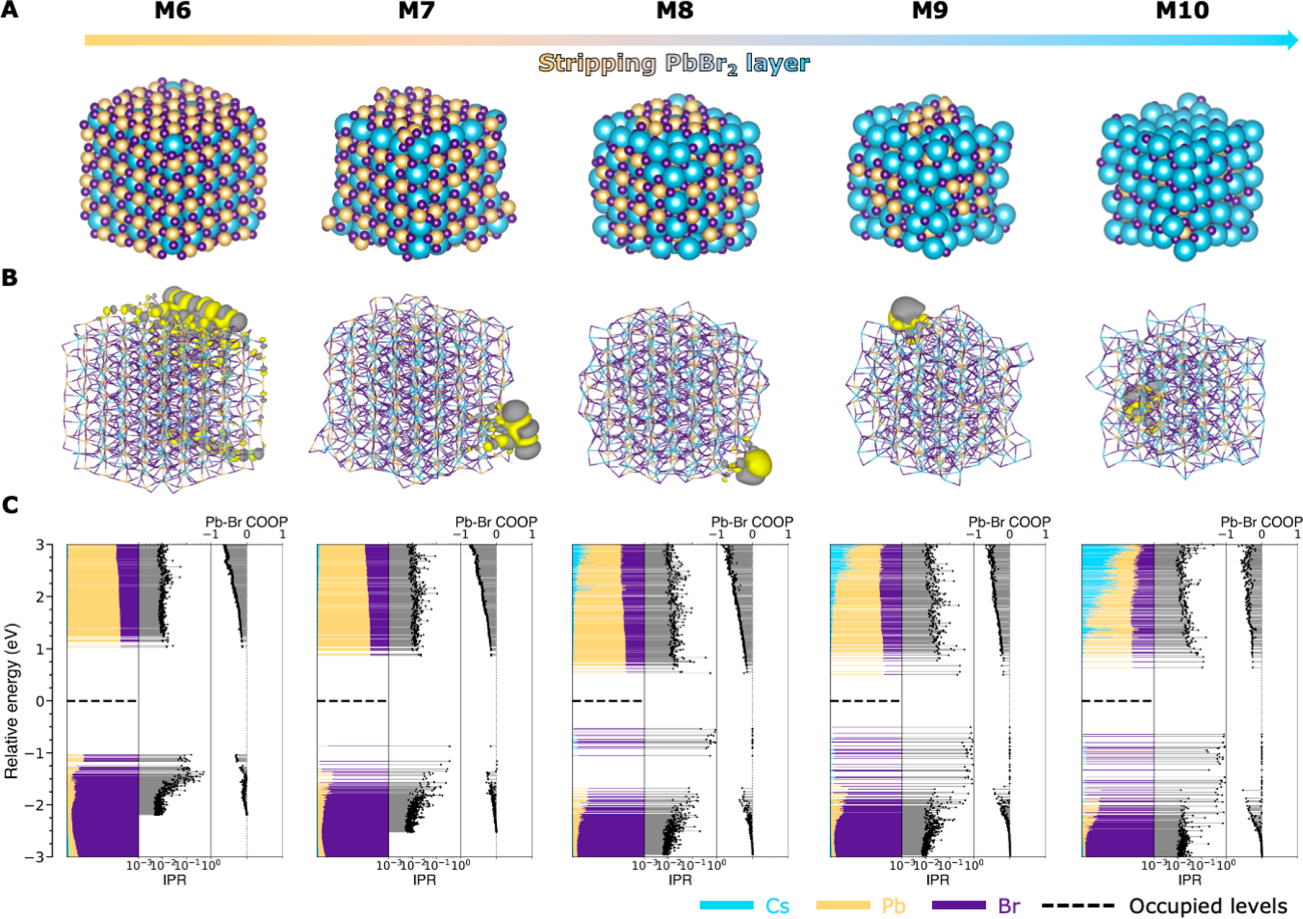
Formation of deep traps upon stripping of the PbBr_2_ layer. (A) Structure, (B) isosurface plot of the HOMO, and (C) density of states (DOS), inverse participation ratio (IPR), and crystal orbital overlap population (COOP) of each model upon gradual stripping of the PbBr_2_ layer. As shown in Figure S2 (models M1–M5), gradual stripping of the outer CsBr layer of our CsPbBr_3_ model system does not create any trap states in the bandgap. However, upon removal of the underlying PbBr_2_ layer (models M6–M10, shown here), deeper states start to appear. From model M8 onward, multiple deep traps, localized on surface Br^–^, are present.

As reported previously,^22^Figure S2 shows that, although removal of the CsBr layer leads to more localized levels (i.e., with a higher IPR) near the VB edge, no deep traps are created, which is in line with the concept of defect tolerance. Upon removal of the PbBr_2_ shell, Br^–^-localized levels start to appear in model M7 (with 25% of the PbBr_2_ removed). As predicted by Figure 1, these Br^–^ levels still largely lie at the VB edge (see Figure S3 for details). However, upon removal of 50% of the PbBr_2_ shell in model M8, many highly localized trap states (IPR ≈ 1) appear in the middle of the bandgap, as shown in Figure 2C. Inspection of the shape of the trap states (Figure 2B, see Figure S4 for more details) and the COOP analysis (COOP ≈ 0, see Figure 2C) reveals that these states are formed by the nonbonding p orbitals of surface Br^–^. Model M8 contains five such Br^–^ sites, each of which creates three trap states with its orthogonal p orbitals, leading to a total of 15 deep traps in Figure 2C.

The models shown in Figures 2 and S2 are constructed by removing CsBr or PbBr_2_ units first from corners and edges, since they possess the lowest binding energy. In reality, removal of PbBr_2_ can lead to a great number of surface compositions. To test that the occurrence of deep traps localized on Br^–^ ions is not specific to the structure of model M8 in Figure 2, we performed additional calculations where PbBr_2_ moieties are either removed from the middle of the facets (Figure S5, models M8-ii and M8-iii) or randomly removed (Figure S5, models M8-iv and M8-v). The observation from these additional calculations is that the formation of deep Br^–^ traps can be generalized to many different PbBr_2_ configurations, as long as Br^–^ ions with only Cs^+^ neighbors are present. Changes in the surface configuration lead to changes in the total energy, but the creation of a Br^–^ trap does not necessarily lead to a significant increase of the energy of the system. Due to the dynamic nature of NC surfaces, many of these configurations will be sampled at room temperature, including configurations that expose uncoordinated surface Br^–^ ions that form deep traps.

Clearly, these trap states are not consistent with the picture of defect tolerance expounded in the first paragraph, which would expect the nonbonding p orbitals to lie in or close to the VB. This suggests that the traditional picture of defect tolerance is incomplete. This picture assumes that the energy of a molecular orbital (MO) only depends on the interaction (be it bonding, antibonding, or nonbonding) between AOs. However, the energy of an AO can also be significantly influenced by its surroundings. For example, crystal field theory describes how the electrostatic field created by the surrounding ligands lifts the degeneracy of d orbitals in metal complexes.^30^ We therefore hypothesize that the appearance of deep trap states can be explained by including the effects of the local electrostatic potential in the description of defect tolerance.

To test this hypothesis, we now take a closer look at model M8, as this is the first model with multiple deep traps. In Figure 3A-ii, we plot the potential energy (in eV), as generated by both the nuclei and electrons, of an electron at the surface of model M8. A blue color in Figure 3A-ii corresponds to a low potential energy, while a red color indicates a high potential energy. Figure 3A-ii shows that the potential energy is significantly higher at five points on the NC surface. These points correspond to the location of the aforementioned five surface Br^–^ sites that are responsible for the 15 deep traps in Figure 3A-iii. What sets these five Br^–^ apart from the other Br^–^ ions in the NC is that they have no direct bonds to Pb^2+^. Instead, their nearest neighbors solely comprise Cs^+^, with which there is little interaction. Whereas all other Br^–^ sites in the bulk and on the surface are stabilized by Pb^2+^, these five surface Br^–^ sites in Figure 3A-ii can practically be seen as loose Br^–^ in vacuum. The absence of Pb^2+^ neighbors means that they experience a significantly higher local potential energy, which pushes their AOs from the VB into the bandgap. The same trends are observed for the models with different PbBr_2_ configurations in Figure S5. There, moving a Br^–^ away from its Pb^2+^ neighbors is also found to significantly raise the potential energy around the Br^–^ ion, thus creating deep traps.

**Figure 3:**
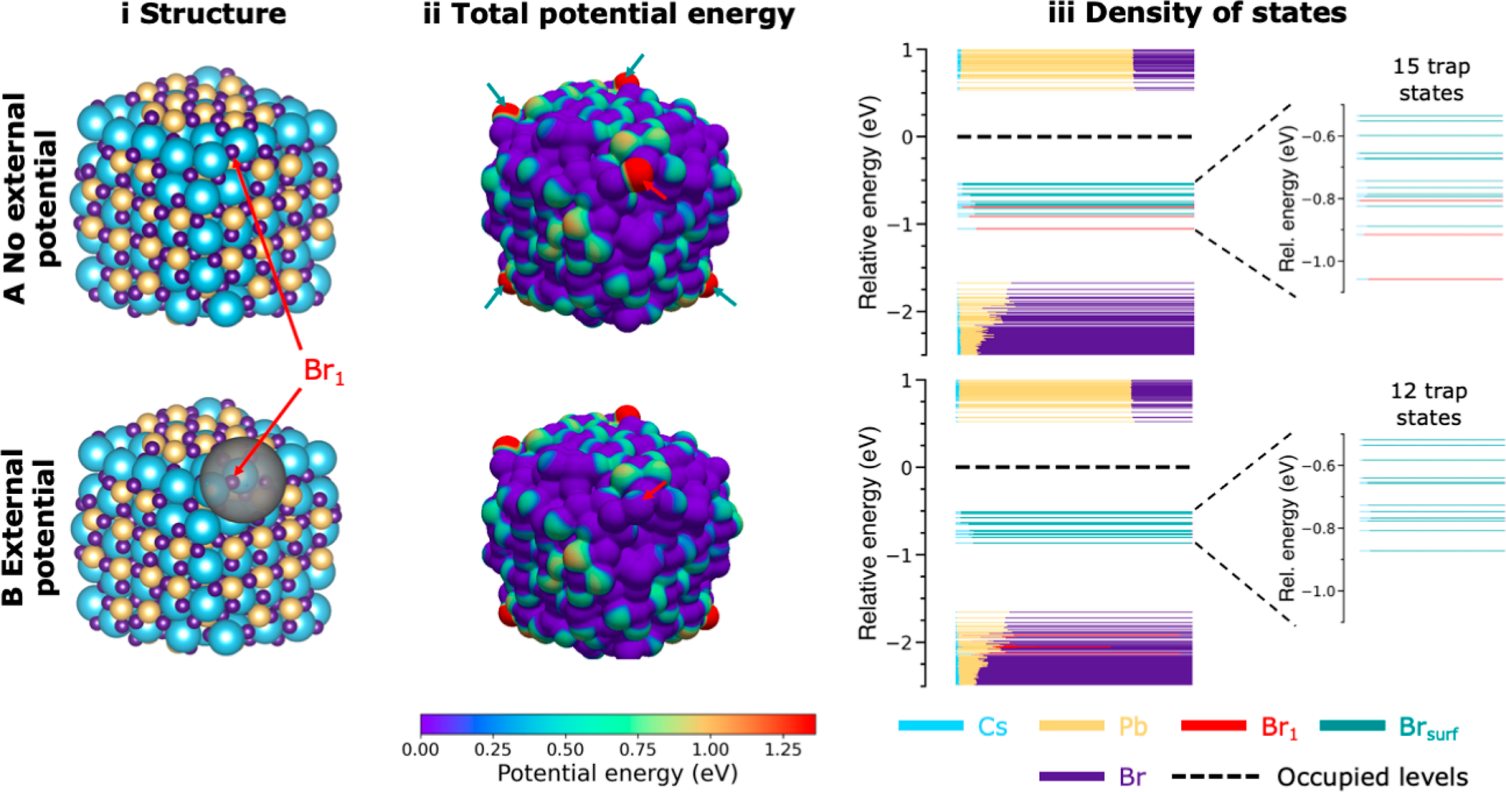
Effect of local potential on the energy of trap states (A) without and (B) with the application of an external potential. (i) Structure of model M8 and the location of the applied external potential around one of the five Br^–^ sites that give a deep trap, indicated as Br_1_. (ii) Total potential energy (i.e., the electrostatic potential generated by the nuclei and electrons plus the external potential, see the Supporting Information for details) of an electron at the NC surface (in eV). Blue colors correspond to a low potential energy, while red indicates a high potential energy. The location of Br_1_ is indicated by the red arrow. The turquoise arrows indicate the other four Br^–^ sites that give rise to deep trap states. (iii) DOS, showing the contribution of the surface Br^–^ to the deep traps. Application of an external potential shifts the states from Br_1_ into the VB, leaving 12 instead of 15 traps in the bandgap.

This reasoning implies that it should also be possible to push deep trap states back into the VB by changing the local potential. In Figure 3B-i we apply an artificial external stabilizing potential (see the Supporting Information for computational details) around one specific Br^–^ (indicated in red as Br_1_ in Figure 3). As shown in Figure 3B-ii, this lowers the potential energy around Br_1_ and consequently pushes the states related to Br_1_ into the VB, leaving 12 instead of 15 deep traps (see Figure 3B-iii). Figure S6 shows that by varying the magnitude of the external potential, the energy of the trap states can be shifted across the bandgap.

These results clearly show that the position of localized MOs depends both on (the absence of) bonds formed with neighboring atoms, as expressed by Figure 1, and on the local electrostatic environment. This conclusion does not depend on the exact surface configuration, but holds generally for undercoordinated Br^–^ ions on the surface, as similar results are obtained on various other surface compositions (see Figure S5). Therefore, one can distinguish two main pathways via which traps may be passivated: (1) the covalent binding of ligands to the surface to split nonbonding trap states and (2) the electrostatic interaction between the NC surface and electrolytes that do not covalently bind to the surface but influence the energy of surface-localized MOs via ionic interactions. In the Supporting Information we show in two ways how traps can be removed through the electrostatic interaction with charges that do not bind covalently to the surface. In Figure S7, we created a core/shell structure, where a CsPbBr_3_ core is surrounded by Cs^+^, Pb^2+^, and Br^–^ like charges to mimic the perovskite bulk potential at the surface of the core. Although these charges do not bind covalently to the surface, Figure S7 shows that the bandgap has become completely trap free, with both the VB and CB edge delocalized over the NC. In Figure S8, we show that states localized on a Br^–^ ion can be removed from the bandgap by addition of a nearby proton (H^+^)-like charge.

In conclusion, we have used DFT calculations to show that the general picture of defect tolerance in cesium lead halide perovskite NCs is incomplete and that the local environment of trap states should also be considered. Br^–^ sites on the surface can experience such a different local potential compared to the bulk that their nonbonding orbitals are pushed into the bandgap and form deep trap states. These results not only give insight into the formation of traps in perovskites but also provide novel approaches for removing these states from the bandgap.

## Supporting Information Available

Computational methods, details of CsPbBr_3_ model system, detailed description of ligand stripping procedure, details of Br^–^ traps in models M7 and M8, effect of stripping the PbBr_2_ layer in different configurations, shifting trap levels by varying the external potential, electrostatic passivation of traps (PDF)
