# Supplementary material for: Limits of Defect Tolerance in Perovskite Nanocrystals: Effect of Local Electrostatic Potential on Trap States
Source: J Am Chem Soc. 2022 Jun 14;144(25):11059–63. doi: 10.1021/jacs.2c02027 (PMC9247979; doi:10.1021/jacs.2c02027)
Supplement: Supplementary file 1 — ja2c02027_si_001.pdf [file ja2c02027_si_001.pdf]

Supporting Information

Limits of Defect Tolerance in Perovskite Nanocrystals: Effect of Local Electrostatic Potential on Trap States

Indy du Fossé,[†] Jence T. Mulder,[†] Guilherme Almeida,[†] Anne G. M. Spruit,[†] Ivan Infante,[§] Ferdinand C. Grozema,[†] Arjan J. Houtepen[†,*]

[†] Optoelectronic Materials Section, Faculty of Applied Sciences, Delft University of Technology, Van der Maasweg 9, 2629 HZ Delft, The Netherlands

[§] Department of Nanochemistry, Istituto Italiano di Tecnologia, Via Morego 30, 16163 Genova, Italy

Contents

Computational methods 2
Details of $CsPbBr_3$ model system 3
Detailed description of ligand stripping procedure 4
Details of Br^- traps in models M7 and M8 6
Effect of stripping the $PbBr_2$ layer in different configurations 8
Shifting trap levels by varying the external potential 10
Electrostatic passivation of traps 11

Computational methods. Geometry optimizations were carried out at the DFT level with a PBE exchange-correlation functional[1] and double-ζ basis set, as implemented in the CP2K quantum chemistry software package.[2] Relativistic effects were taken into account using effective core potentials. Due to the large size of the systems, spin-orbit coupling was not included.

The inverse participation ratio (IPR) and crystal orbital overlap population (COOP) were computed for the 500 highest occupied and 500 lowest unoccupied orbitals of each model. The IPR was calculated via $IPR_i = \sum_\alpha |P_{\alpha,i}|^4 / \left(\sum_\alpha |P_{\alpha,i}|^2\right)^2$. Here, IPR_i is the IPR of the ith molecular orbital (MO) and $P_{\alpha,i}$ the weight of the MO on atom α. The IPR ranges from 1/N (with N the total number of atoms in the system) for a completely delocalized MO, to 1 for complete localization on one atom. The COOP between Pb and Br was computed through $COOP_i^{Pb-Br} = \sum_{n \in Pb} \sum_{m \in Br} S_{nm} \cdot c_{n,i} \cdot c_{m,i}$. Here, $COOP_i^{Pb-Br}$ is the COOP of the ith MO and S_{nm} the overlap between atomic orbitals (AOs) n and m, with coefficients $c_{n,i}$ and $c_{m,i}$, respectively. n_i loops over the atomic orbitals (AOs) of all Pb atoms, m_i loops over the AOs of all Br. A negative COOP value indicates that an MO is of an overall anti-bonding character, while positive COOP values indicate an overall bonding MO. COOP values close to zero are indicative of a non-bonding MO. Both the IPR and the COOP were calculated using the workflows implemented in the Nano-QMFlows package.[3]

The external potential, mentioned in the main text during the discussion of Figure 3, was applied using the “EXTERNAL_POTENTIAL” keyword in CP2K. We introduced a Gaussian potential, centered 3.3 Å away from Br_1 (see Figure 3 in the main text), and described by $V_{ext} = A \cdot \exp\left(-\frac{(x-39.32286428)^2 + (y-8.17028545)^2 + (z-34.22436985)^2}{2 \cdot 3.0^2}\right)$. Here, A is the maximal amplitude of the external potential (which we varied between -0.1 and 0.1 Ha in Figure S6) and x, y, z are the spatial coordinates in Å. This potential is added as an extra term to the Hamiltonian. As a result, a *positive* external potential (*i.e.*, $A > 0$) *lowers* the energy of the electrons.

The total potential energy experienced per MO (as plotted in Figures S3C, S4C and S6A) was calculated via $V_{total,i} = \frac{\langle \psi_i | V_{ion} - V_{ext} | \psi_i \rangle}{\langle \psi_i | \psi_i \rangle}$, where V_{ion} is the electrostatic potential generated by all the electrons and nuclei, V_{ext} the external potential (if applied) and ψ_i the wavefunction of the ith MO. Note that we subtract V_{ext} from V_{ion} to correct for the opposite sign of V_{ext}.

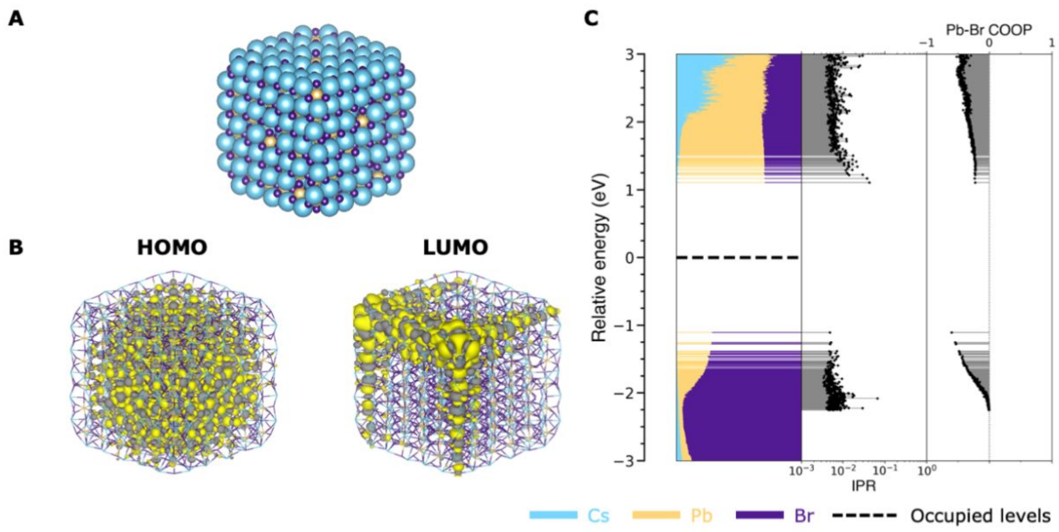

Figure S1. Details of the $Cs_{324}Pb_{216}Br_{756}$ model system M1. (A) Structure, (B) HOMO and LUMO and (C) density of states (DOS), IPR and COOP of the NC.

Ligand stripping. Following the method by Bodnarchuk *et al.*,[4] we remove the outer CsBr layer of the model system M1 (shown in Figure S1) in four consecutive steps, in which we remove 25%, 50%, 75% and 100% of the CsBr units. This is shown in models M2-M5 in the first half of Figure S2. After removal of all CsBr units, the NC is terminated by a layer of $PbBr_2$, with some loose Br^- left on the NC surface. These loose Br^- are removed in model M6. Subsequently, in four consecutive steps, we again remove 25%, 50%, 75% and 100% of the $PbBr_2$ shell (models M7-M10, shown in the second half of Figure S2 on the next page).

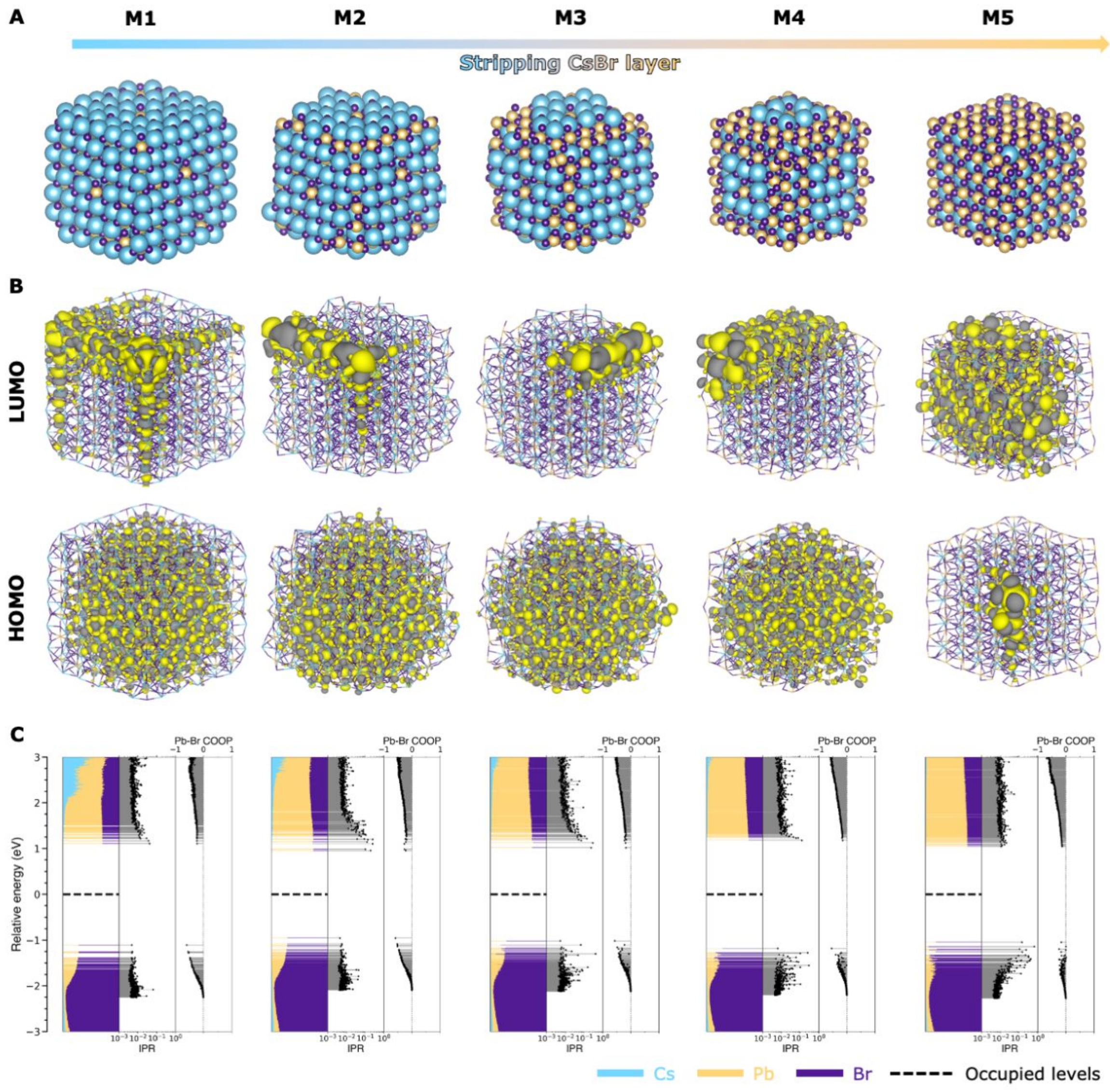

Figure S2. Stepwise removal of CsBr and $PbBr_2$ layers from model system M1. (A) Structure, (B) HOMO and LUMO, and (C) DOS, IPR and COOP of each NC.

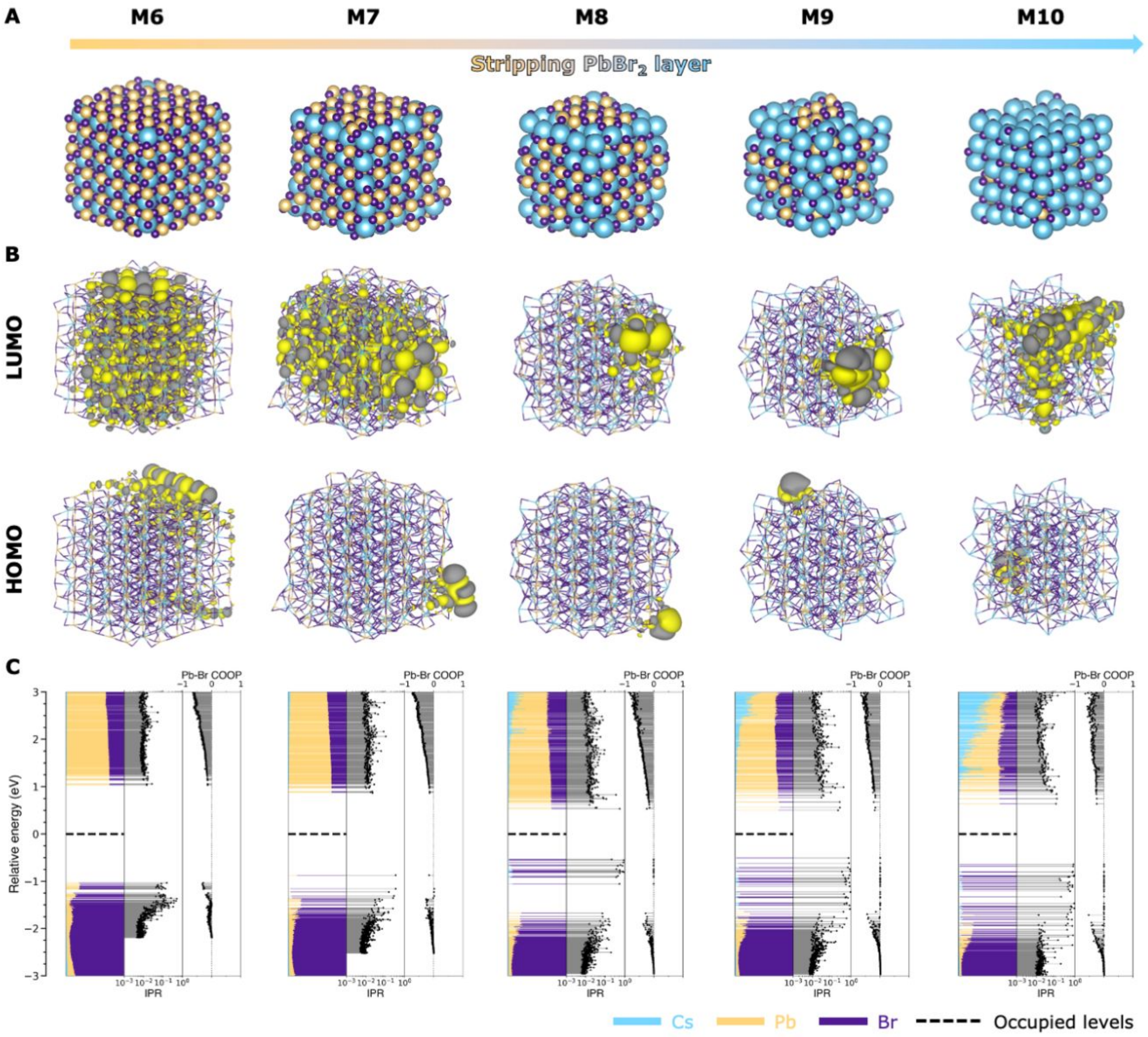

Figure S2 (continued). Stepwise removal of CsBr and $PbBr_2$ layers from model system M1. (A) Structure, (B) HOMO and LUMO, and (C) DOS, IPR and COOP of each NC.

Shallow traps in model M7. As discussed in the main text, removal of 25% of the $PbBr_2$ layer leads to the creation of Br^--localized levels at the VB edge, which are shown in more detail in Figure S3. The highest five occupied levels are located on the $PbBr_2$ unit indicated in turquoise in Figure S3A. In the HOMO, there is a slight anti-bonding interaction between the Br^- and Pb^{2+}, as indicated by the small Pb-contribution in the DOS and the negative COOP-value in Figure S3C. The four levels below the HOMO (*i.e.*, HOMO-1 through HOMO-4) do not interact with Pb^{2+}, as evinced by the COOP values of ~0. However, as can be seen in Figure S3B, the two Br^- ions do interact with each other, forming π and π* bonds. As the π- and π*-like orbitals lie very close in energy, the Br^--Br^- interaction is expected to be small. Since the trap levels are close to the VB edge, these results are still in agreement with the concept of defect tolerance as discussed in the main text. However, further stripping of the $PbBr_2$ layer creates Br^--localized levels that do lie in the middle of the bandgap (see Figure S4 and the main text).

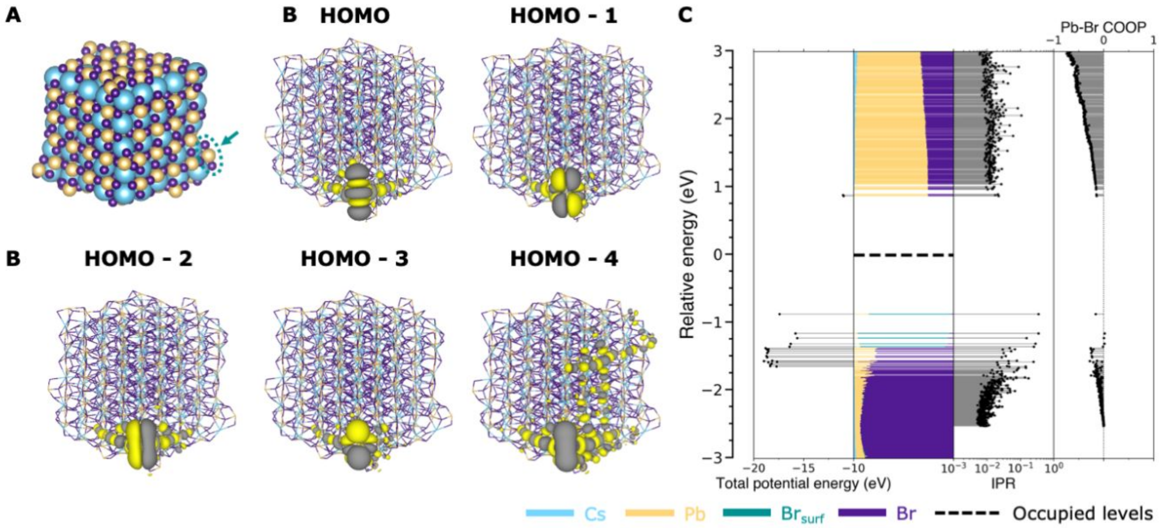

Figure S3. Details of the shallow traps formed in model M7. (A) Structure of model M7. The five highest occupied levels are localized on the two Br^- ions indicated by the turquoise arrow. (B) Shape of the five highest occupied MOs. (C) DOS, IPR and COOP of the NC. The contribution of the two Br^- ions are shown in turquoise. The leftmost panel shows the total potential energy for each MO around the bandgap (see Computational methods for details).

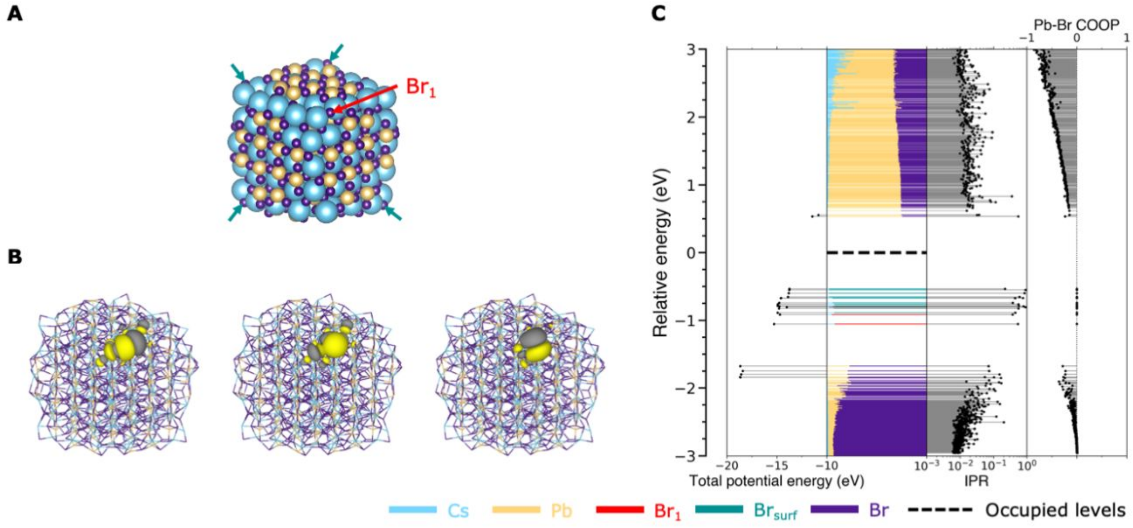

Figure S4. Details of the deep traps formed in model M8. (A) Structure of model M8. The deep traps are localized on five Br^- ions. Four are indicated by the turquoise arrows, while Br_1 is indicated in red (as also done in Figure 3 in the main text). (B) Shape of the three traps localized on Br_1. Each Br^- site creates three trap states, formed by three orthogonal p-orbitals. (C) DOS, IPR and COOP of the NC. The contribution of the five Br^- ions are shown in turquoise and red. The leftmost panel shows the total potential energy for each MO around the bandgap (see Computational methods for details).

Effect of removing $PbBr_2$ in different configurations. As discussed in the main text, the occurrence of deep Br^- traps in model M8 is not linked to the specific structure of M8. Instead, it can be generalized to many different surface configurations, as long as Br^- ions with only Cs^+ neighbors are present. To show Br^- traps are not unique to model M8, we demonstrate in Figure S5 that removing 50% of the $PbBr_2$ moieties from different surface sites can also lead to the formation of deep traps.

Model M8, which is used in the main text (and also plotted in Figure S2), is shown in the leftmost column. Following the approach of Bodnarchuk *et al.*,[4] $PbBr_2$ moieties were removed first from the corners and edges of the NC. In model M8-ii, $PbBr_2$ was first removed from the center of each facet. As shown in Figure S5A, this surface configuration is less favorable, increasing the total energy by 1.0 eV (compared to model M8). Moreover, all Br^- are coordinated to Pb^{2+}, so that no traps are present in Figure S5D, confirming our interpretation of the traps as arising from Br^- with no coordination to Pb^{2+}. Moving one Br^- from model M8-ii away from its Pb^{2+} neighbors results in structure M8-iii (the red circle indicates which Br^- has been moved). In line with the results shown in the main text, this Br^-, which now only has Cs^+ neighbors, experiences a significantly higher potential energy (see Figure S5C). As also described in the main text, this leads to the formation of three deep traps, which are indicated in turquoise in Figure S5D. Yet, note that displacing the Br^- increases the energy of the system by 0.4 eV, which makes the formation of this specific trap energetically unfavorable. In model M8-iv, surface $PbBr_2$ moieties have been removed from random positions. No traps are found due to the coordination of all surface Br^- to Pb^{2+}. Again, moving one Br^- (indicated by the orange circle) away from Pb^{2+} causes the Br^- to experience a higher local potential energy, leading to the formation of three traps. However, in contrast to model M8-iii, there is only a negligible change in the total energy, indicating that both systems are roughly equally likely to be sampled at room temperature.

These results show that, although model M8 as used in the main text is not the lowest energy configuration, the formation of deep Br^- traps can be generalized to many different $PbBr_2$ configurations, as long as Br^- ions with only Cs^+ neighbors are present. Changes in the surface configuration can also lead to changes in the total energy, but the creation of a Br^- trap does not necessarily lead to a significant increase of the energy of the system. Hence, thermal motion will cause the NC to sample a great number of surface compositions, with varying numbers of Br^- traps.

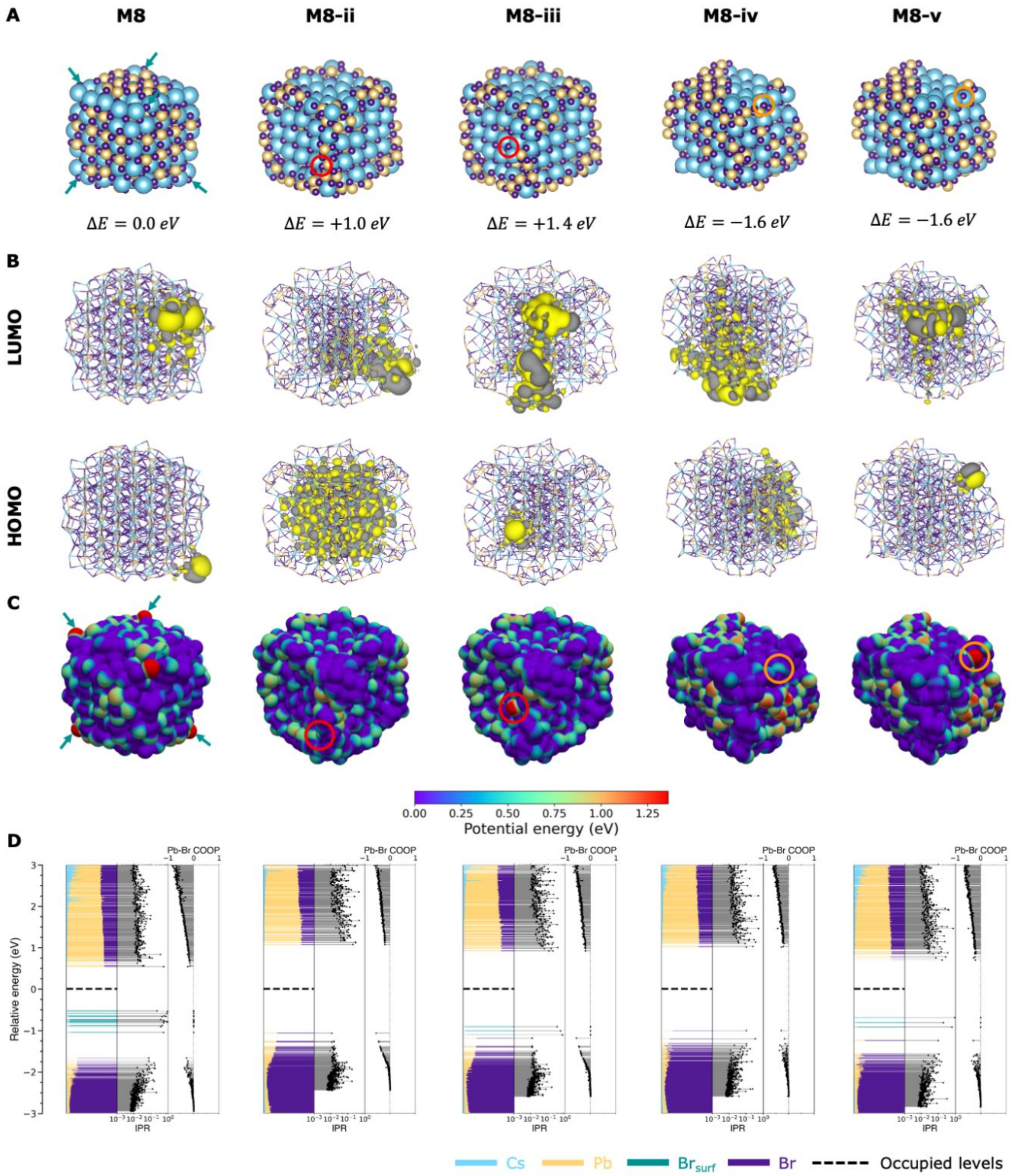

Figure S5. Effect of stripping the $PbBr_2$ layer in different configurations. Model M8 (used in the main text): $PbBr_2$ first removed from corners and edges. Model M8-i: $PbBr_2$ first removed from the middle of each facet. Model M8-iii: Same as model M8-ii, but one Br^- has been moved (see red circle). Model M8-iv: $PbBr_2$ removed from random surface sites. Model M8-v: Same as Model M8-iv, but one Br^- has been moved (see orange circle). (A) Structure and energy (relative to model M8 as used in the main text), (B) HOMO and LUMO, (C) the potential energy (in eV) of an electron at the NC surface, and (D) DOS, IPR and COOP of each NC. The contribution of surface Br^- ions (which are either indicated by the turquois arrows or the red or orange circles) is given in turquois.

Shifting energy of traps. As discussed in the main text and shown in Figure 3B, application of a stabilizing external potential around Br_1 can push the energy of the traps back into the VB. Figure S6 shows that by varying the magnitude of the applied potential, the traps localized on Br_1 can be shifted through the entire bandgap. Figure S6A plots the energy (relative to the VB edge) of the three traps localized on Br_1 (shown in black, red, and blue) against the total potential energy experienced by those traps (see Computational methods for details). It can be seen that a more negative total potential energy lowers the energy of the trap with respect to the VB, and eventually pushes the trap below the VB edge (as indicated by the negative energy in Figure S6A). Conversely, a less negative total potential energy increases the energy of the traps with respect to the VB, and pushes them towards the CB, as also illustrated in Figure S6B.

The fits in Figure S6A show a strong linear correlation between the energy of the traps vs the VB edge and their total potential energy, with a slope in the range of 0.8-1.0. This suggests that the shift in energy is largely caused by the change in total potential energy, but that small additional effects take place in parallel, leading to a slope of not exactly 1. One significant outlier, in red at a total potential energy of -17 eV, may be explained by the fact that this trap, which lies in the VB, is slightly mixed with other VB levels. This is expected to lead to additional energy effects that break up the linear correlation.

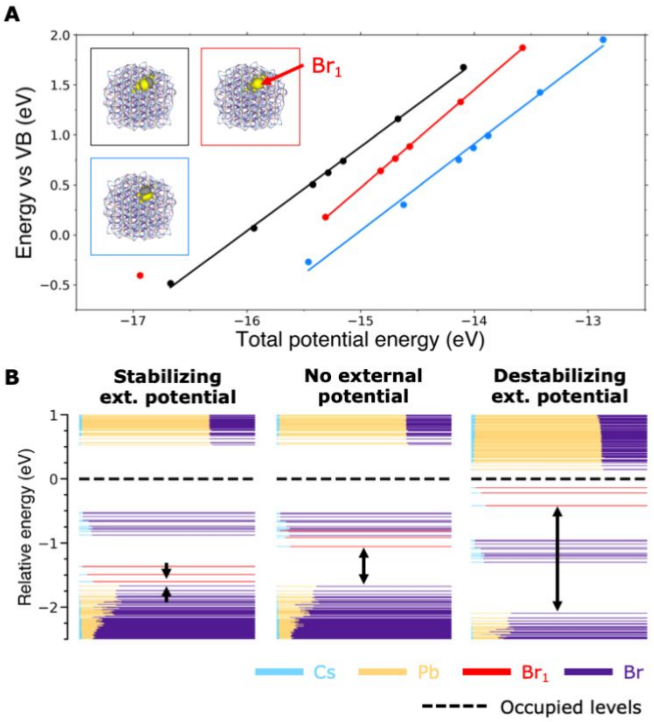

Figure S6. Shifting the energy of the traps located on Br_1 by application of an external potential. (A) Energy of the trap (relative to the VB edge) against the total potential energy, as experienced by the three trap states localized on Br_1. The fits show a linear correlation, with the exclusion of the outlier in red at a total potential energy of -17 eV. (B) DOS, showing the location of the Br_1 traps upon application of a stabilizing ($V_{ext} > 0$, see Computational methods) and destabilizing ($V_{ext} < 0$) external potential.

Electrostatic trap passivation. In order to investigate if deep traps can be removed solely through the electrostatic effect of ligands (*i.e.*, without covalent binding to, and splitting of the non-bonding Br^--orbital), we carried out two types of calculations. In the first, we constructed a sort of core/shell structure, based on model M1, as shown in Figure S7A. Here, the shell (indicated in gray) consists of all the atoms of model M1 that are removed during the stripping procedure (Figure S2) to create model M8. The core (indicated with the standard colors for Cs, Pb and Br) comprises the same atoms that are in model M8. However, note that the coordinates of the core atoms in Figure S7A are not exactly the same as in model M8, since the coordinates of the former are extracted from the geometry optimization of model M1 in Figure S2. As a result, the atoms at the surface of the core (in Figure S7A) still have the same configuration as they had in the bulk of model M1. Carrying out a single point calculation on the core atoms only, as shown in Figure S7D "without shell", indicates that this type of NC termination leads to many trap states and virtually no bandgap.

In the main text, we argued that the different potential experienced at the surface, causes Br^- dangling bonds to end up in the bandgap. By extension, one would expect that the traps in Figure S7D will be pushed out of the bandgap if the potential experienced at the core surface is equal to the potential experienced in the bulk. To simulate this effect, we removed the basis functions and electrons of all the atoms in the shell (gray in Figure S7A), leaving only the effective core potentials (see Computational methods). We corrected the charge of the core potentials so that the atoms in the shell have a charge of Cs^+, Pb^{2+} and Br^-. As a result, the atoms in the shell mimic the perovskite bulk potential at the surface of the core, without forming any covalent bonds with the core surface. Figure S7D shows that addition of this "point charge" shell effectively removes all traps from the bandgap. Figures S7B and S7C show that the band edges are delocalized over the entire core. Note that the MOs do not delocalize over the shell, as these atoms do not have basis functions.

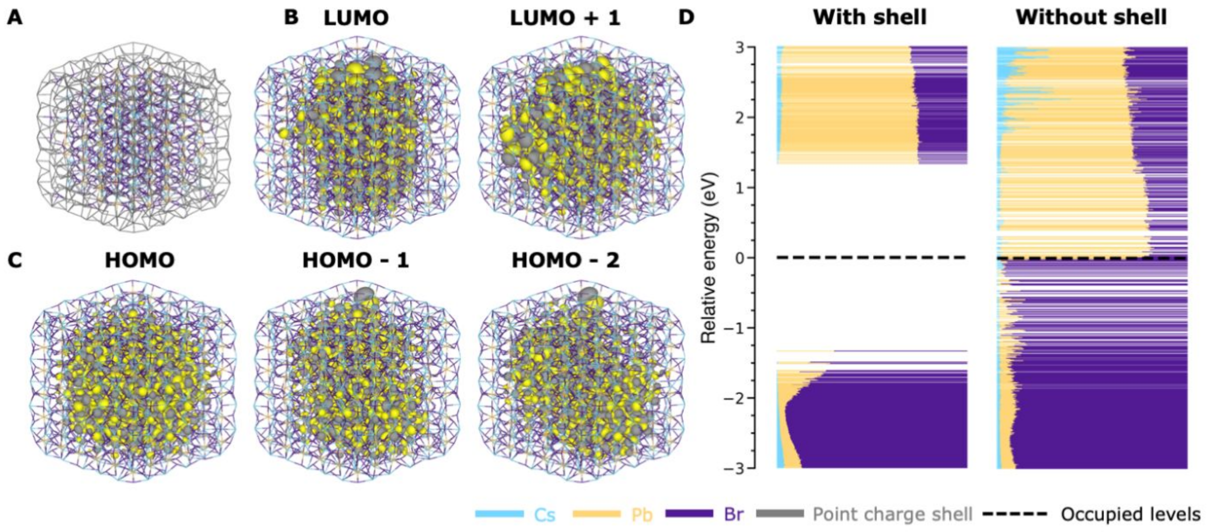

Figure S7. Electrostatic trap passivation by addition of a "point charge" shell. (A) Structure of the core/shell structure. The shell is indicated in gray. (B) Shape of the CB edge levels and (C) the VB edge levels. (D) DOS of the core with and without the shell.

In the second calculation, the traps related to Br_1 were pushed out of the bandgap by placing a proton (H^+)-like charge in the vicinity of Br_1 (see Figure S8A). To ensure no covalent binding to Br_1 takes place, we removed the basis functions of the proton. In addition, we removed the electron of the H^+ by setting the charge of the overall system (*i.e.*, NC + proton) to +1, leaving only the effective core potential of the proton (with a total charge of +1). Figure S8B shows that the addition of the H^+ lowers the potential energy around Br_1, which pushes the trap states on Br_1 back into the VB, as shown in red in Figure S8C. As the H^+ is only placed in the vicinity of Br_1, the other four Br^- sites (indicated in turquoise in Figure S8A) still experience a high potential energy, which leaves their traps in the bandgap.

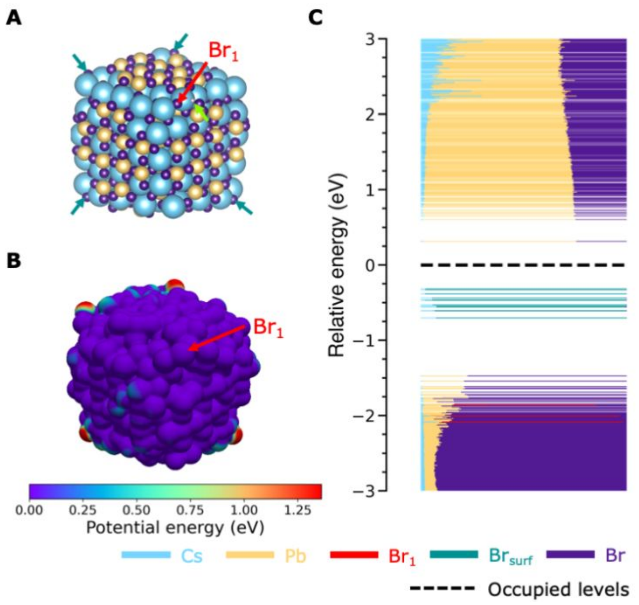

Figure S8. Electrostatic trap passivation by addition of a proton (H^+)-like charge. (A) Location of the H^+ (indicated by the light green arrow), which is placed 6.5 Å away from Br_1 (indicated in red). (B) The potential energy (in eV) of an electron at the NC surface. (C) DOS, showing the contribution of Br_1 and the other four Br^- sites (indicated in turquoise) that give deep traps.

References

1. Perdew, J. P., Burke, K. & Ernzerhof, M. Generalized Gradient Approximation Made Simple. *Phys. Rev. Lett.* **77**, 3865–3868 (1996).
2. Hutter, J., Iannuzzi, M., Schiffmann, F. & VandeVondele, J. cp2k: atomistic simulations of condensed matter systems. *Wiley Interdiscip. Rev. Comput. Mol. Sci.* **4**, 15–25 (2014).
3. Zapata, F., Ridder, L., Hidding, J., Jacob, C. R., Infante, I. & Visscher, L. QMflows: A Tool Kit for Interoperable Parallel Workflows in Quantum Chemistry. *J. Chem. Inf. Model.* **59**, 3191–3197 (2019).
4. Bodnarchuk, M. I., Boehme, S. C., Ten Brinck, S., Bernasconi, C., Shynkarenko, Y., Krieg, F., Widmer, R., Aeschlimann, B., Günther, D., Kovalenko, M. V., *et al.* Rationalizing and Controlling the Surface Structure and Electronic Passivation of Cesium Lead Halide Nanocrystals. *ACS Energy Lett.* **4**, 63–74 (2019).
